# If it's on Facebook, it must be true! Body image and social media

**DOI:** 10.1186/2050-2974-2-S1-P6

**Published:** 2014-11-24

**Authors:** Genevieve Pepin, Natalie Endresz

**Affiliations:** 1 Deakin University, Melbourne, Australia

## 

Literature has identified and supported links between exposure to media such as fashion magazines or television and body image concerns. With social media and networking sites becoming increasingly popular, the influence these media and sites can have on the body image of young persons deserves further investigation. Building on a recent study that revealed that young girls using Facebook had significantly more body image concerns than non-users, this study examines the influence of the use of social networking sites on the body image of 100 university students aged 18-25.

A quantitative design with repeated measures where participants will complete online surveys about their use of social media over 4 months is being implemented. Use of social media, content posted on these sites, responses expected, wished for and obtained will be documented. The Objectified Body Consciousness Scale, the Sociocultural Attitudes Towards Appearance Questionnaire and the Eating Disorders Inventory 3 will be used to identify impacts of using social media and networking sites on body image.

Results of this study will extend existing evidence exploring links between social media and body image concerns and contribute new information to the development of media literacy programs addressing negative body image.

